# Fire Severity Drives Long-Term Divergence of Soil Microbial Communities and Functional Traits in an Alpine Forest

**DOI:** 10.3390/microorganisms14092115

**Published:** 2026-09-21

**Authors:** Xun Li, Wenxi Yang, Yan Zhang, Bin Peng, Xiaolin Li

**Affiliations:** 1 Key Laboratory of Ecological Protection and Characteristic Industry Cultivation in Hengduan Mountain Area of Department of Education and Ganzi Prefecture, Sichuan Minzu College, Kangding 626001, China; xunlichina@163.com (X.L.); y004116@163.com (W.Y.); zhangyanyb@163.com (Y.Z.); 2 Sichuan Institute of Edible Fungi, Sichuan Academy of Agricultural Sciences, Chengdu 610066, China; lixiaolin@scsaas.cn

**Keywords:** fire severity, soil microorganisms, community structure and function, alpine forest, burned site

## Abstract

Increasing wildfire severity under climate change may alter the trajectory of belowground recovery in alpine forests, yet its long-term effects on soil microbial communities remain poorly understood. Here, we examined soil physicochemical properties and microbial communities eight years after fire across unburned, low-, moderate-, and high-severity fire sites in an alpine forest of western Sichuan, China. Fire severity was associated with persistent divergence at depths of 0–10 cm, with significant changes in soil organic carbon (SOC), soil water content (SWC), available nitrogen (AN), and microbial biomass. High-severity fire strongly reduced AN and available phosphorus (AP), whereas moderate-severity fire maintained the highest SOC and SWC. Bacterial alpha diversity remained relatively stable, whereas fungal richness declined markedly under high-severity fire, indicating greater fungal sensitivity to fire disturbance. Fire severity explained 57.35% and 87.32% of bacterial and fungal community variation, respectively, while structural equation modeling indicated that microbial responses were primarily mediated by changes in nutrient availability, soil moisture, and microbial biomass. Functionally, microbial communities shifted from resource-acquisition strategies under low-severity fire toward potential stress-tolerant and saprotrophic-dominated strategies under high-severity fire. Moderate-severity fire enhanced bacterial network complexity and increased the abundance of specific mycorrhizal taxa (e.g., *Russula*), whereas high-severity fire promoted saprotrophic and stress-resistant taxa such as Actinobacteriota and *Penicillium*, while suppressing mycorrhizal groups. Overall, fire severity acted as a persistent environmental filter that reorganized microbial community structure and functional strategies of the topsoil, suggesting that increasing fire severity may delay belowground recovery and weaken ecosystem resilience in alpine forests.

## 1. Introduction

Driven by climate change and increasing anthropogenic pressures, the frequency, extent, and severity of wildfires have increased substantially worldwide in recent decades. Fire has consequently emerged as a major driver of ecosystem dynamics in forested landscapes worldwide [1,2,3]. Understanding how fire severity regulates ecosystem recovery remains a major challenge in global change ecology [4]. Soil microorganisms regulate critical belowground processes, including organic matter decomposition, nutrient cycling, and plant nutrient acquisition [5]. Following fire, alterations in soil thermal regimes, moisture availability, nutrient pools, and vegetation cover restructure microbial habitats and modify community composition and ecological functions [6]. Given that microbial communities mediate soil fertility recovery and vegetation re-establishment, their responses to fire disturbance contribute substantially to ecosystem recovery and resilience. Alpine forests are particularly important because they provide essential ecosystem services, including water regulation and carbon storage, yet their recovery after fire is constrained by low temperatures and limited nutrient availability.

Fire not only alters microbial diversity and community structure but also reshapes their ecological strategies through strong environmental filtering [7]. During combustion, large amounts of soil organic matter are volatilized or transformed into pyrogenic carbon, while elevated temperatures reduce soil moisture, disrupt aggregate structure, and modify nutrient availability [8]. Collectively, these changes create a resource-limited and highly stressful environment that selects for microbial taxa characterized by enhanced stress tolerance, metabolic flexibility, and efficient nutrient acquisition strategies [9,10]. Consequently, post-fire microbial communities often undergo functional reorganization, shifting from strategies dominated by rapid growth and resource exploitation toward strategies emphasizing survival maintenance and resource conservation. For instance, bacterial communities tend to enhance metabolic pathways associated with stress response, carbon-use efficiency, and dormancy regulation, whereas fungal communities often shift from dominance by ectomycorrhizal fungi to saprotrophic taxa capable of utilizing fire-derived organic substrates [6]. These transitions reflect adjustments in microbial resource utilization strategies and energy allocation patterns, ultimately influencing ecosystem-scale carbon and nutrient cycling processes after fire disturbance [11].

Accumulating evidence indicates that fire severity exerts a strong influence on post-fire microbial dynamics. Compared with low-severity fires, high-severity fires typically result in greater loss of organic matter, more pronounced alterations in soil physicochemical properties, and more extensive disruption of microbial habitats. Under such conditions, bacterial and fungal communities exhibit distinct response trajectories: fungi are generally more sensitive due to the vulnerability of hyphal networks and mycorrhizal structures, whereas bacteria often recover more rapidly owing to their shorter life cycles and higher environmental adaptability [12]. Moreover, the impacts of fire on soil microbial communities can be long-lasting. Several studies have shown that even years after fire events, microbial communities remain significantly different from those in unburned reference sites, suggesting persistent effects on belowground ecosystem processes and nutrient cycling [13]. Post-fire microbial responses involve not only taxonomic shifts but also functional reorganization, including alterations in decomposition pathways, nutrient acquisition strategies, and interspecific microbial interactions.

Despite these advances, important knowledge gaps remain. Most previous studies have focused primarily on short-term microbial responses within the first few years following fire, while the long-term effects of fire severity on microbial community succession remain poorly understood [14]. This gap is especially pronounced in alpine forests, where long-term post-fire microbial succession has rarely been examined, despite the urgent need to inform restoration of these fragile and slowly recovering ecosystems. In particular, it remains unclear whether fire severity can induce persistent niche differentiation among microbial communities many years after disturbance, and how post-fire soil environmental changes regulate the process and extent of microbial functional reassembly [10,11]. Addressing these questions is especially critical in alpine forest ecosystems, where low temperatures and slow nutrient turnover may prolong ecosystem recovery and amplify legacy effects in belowground processes [15]. The alpine forests in western Sichuan, located on the eastern margin of the Qinghai–Tibetan Plateau, provide an ideal natural laboratory for investigating these mechanisms [16]. As an important ecological security barrier in southwestern China, this region plays a key role in water conservation and carbon storage; however, it has experienced increasingly frequent wildfire activity under recent climate warming [15,17]. Given their ecological importance and slow recovery, thorough investigation of post-fire microbial responses in these alpine forests is essential for developing effective restoration strategies.

Based on these knowledge gaps, we hypothesized that: (1) increasing fire severity is associated with persistent divergence in bacterial and fungal community composition, with fungal communities exhibiting greater sensitivity to fire disturbance than bacterial communities; (2) increasing fire severity promotes shifts in microbial functional strategies from resource acquisition toward stress tolerance and decomposition processes; and (3) fire-induced changes in microbial communities are mediated, at least partly, by alterations in soil moisture and nutrient availability. To test these hypotheses, we conducted a comprehensive field investigation eight years after fire disturbance across unburned, low-, moderate-, and high-severity burned alpine forest soils. By integrating analyses of soil physicochemical properties, microbial community composition, predicted functional potential, and environmental drivers, we investigated how fire severity influences long-term microbial succession and belowground recovery processes in alpine forest ecosystems. By examining an understudied alpine system and evaluating eight-year post-fire legacy effects across a severity gradient, this study provides insights into the processes underlying belowground microbial responses and their implications for post-fire ecosystem management.

## 2. Materials and Methods

### 2.1. Study Area

**Figure 1:**
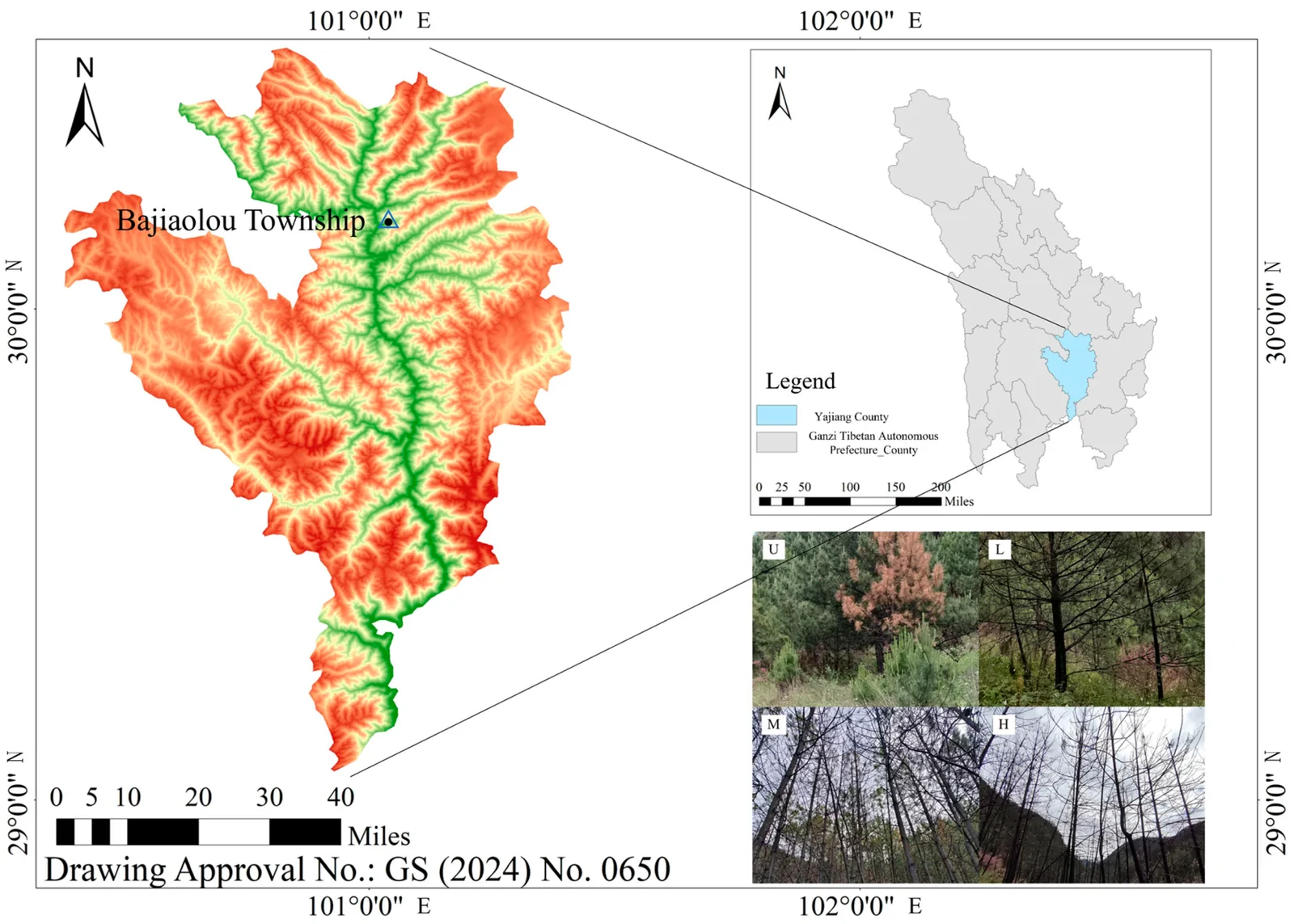
Location of the study area and fire severity sampling plots. U, unburned control; L, low severity; M, moderate severity; and H, high severity.

The study area is located in Gengjue Village, Bajiaolou Township, Yajiang County, Ganzi Tibetan Autonomous Prefecture, Sichuan Province, China (101°07′–101°16′ E, 30°05′–30°14′ N; Figure 1). The region is characterized by a continental climate, with a mean annual temperature of 11.1 °C, mean annual precipitation of 822.2 mm, mean annual potential evaporation of approximately 1000–1400 mm, and mean annual sunshine duration of 1770.7 h. A forest fire occurred in this region on 19 February 2018, affecting an area of approximately 100 ha. During the sampling period, strict fire control measures were implemented in the study sites, and the entry of any open flame was prohibited to prevent secondary burning.

Following the fire, residual biomass within the burned area primarily consisted of standing dead trees and pyrogenic carbon deposits on the soil surface. The dominant tree species in the region is *Pinus densata*, and the soil type is classified as brown soil [18], corresponding to Cambisols in the World Reference Base for Soil Resources (WRB) [19]. Based on the proportion of dead standing trees and the degree of canopy scorching, fire severity was classified into three levels: low, moderate, and high (Table A1) [20].

### 2.2. Plot Establishment and Soil Sampling

In July 2025, the burned *Pinus densata* forest area described above was selected as the study site. Three 20 m × 20 m plots were established for each fire severity level (low, moderate, and high). In addition, three unburned control plots were established in adjacent intact *P. densata* forests. Plots were designated as U (unburned control), L (low severity), M (moderate severity), and H (high severity). All plots were located on mid-slopes at high elevations above 2800 m, with predominantly semi-shaded to semi-sunny exposures. No obvious gullies, landslides, or large depressions were observed within the plots. The burned plots contained standing dead trees and shrub stumps, which helped mitigate wind and water erosion of the soil surface. All burned plots were affected by the same major forest fire, and no other forest fires occurred in the study area within the 8 years following the event. Within each treatment group, the distance between replicate plots was at least 50 m, and they were distributed across different microtopographic locations within the burned area to ensure spatial independence; plots within the same fire severity category were generally consistent in terms of elevation, slope aspect, and soil texture. The basic characteristics are provided in Table A2.

Within each plot, soil samples were collected using a five-point sampling strategy. After removing surface litter, soil profiles were excavated. Undisturbed soil samples from the 0–10 cm layer were collected using a stainless-steel ring core sampler (100 cm^3^) for physical property analysis. Concurrently, bulk soil samples from the same depth were collected, placed in sterile polyethylene bags, and transported to the laboratory under cooled conditions (−20 °C) for further analysis. In the laboratory, visible roots and gravel were removed. Each sample was homogenized and divided into two subsamples: one for soil chemical analyses, and the other was stored at −80 °C for subsequent microbial community analysis.

### 2.3. Analytical Methods

#### 2.3.1. Soil Physicochemical Properties

Soil physicochemical properties were determined following the methods of Bao [21]. Soil pH was measured using a potentiometric method, and electrical conductivity (EC) was determined using a conductivity meter. Soil water content (SWC) was measured by the gravimetric oven-drying method. Available phosphorus (AP) was quantified using sodium bicarbonate extraction followed by molybdenum–antimony anti-colorimetric spectrophotometry. Available potassium (AK) was determined using ammonium acetate extraction and flame photometry. Ammonium nitrogen (NH_4_^+^–N, AN) and nitrate nitrogen (NO_3_^−^–N, NN) were measured using potassium chloride extraction followed by colorimetric analysis.

Soil aggregate stability was assessed using the wet-sieving method, and was expressed as mean weight diameter (MWD) and geometric mean diameter (GMD) [22]. Soil organic carbon (SOC) was measured using a total organic carbon (TOC) analyzer. Water-soluble organic carbon (WSOC) was determined by aqueous extraction followed by TOC analysis [23]. Easily oxidizable organic carbon (EOC) was measured using potassium permanganate oxidation and spectrophotometric analysis [24].

#### 2.3.2. Soil Microbial Community Analysis

Soil microbial communities were characterized using high-throughput sequencing (Novogene Co., Ltd., Beijing, China). Briefly, PCR amplification was performed, followed by library construction and paired-end sequencing on an Illumina NovaSeq platform. The V3–V4 region of the bacterial 16S rRNA gene and the ITS1 region of fungi were amplified (primers CCTAYGGGRBGCASCAG,GGACTACNNGGGTATCTAAT and CTTGGTCATTTAGAGGAAGTAA,GCTGCGTTCTTCATCGATGC, respectively) to determine microbial diversity [25]. Raw reads were demultiplexed, quality-filtered, paired-end merged, and chimeras were removed on Novogene Cloud platform. Bioinformatic processing was based on QIIME2 (version 202202): FLASH (v1.2.11) for read merging, fastp (v0.23.1) for quality filtering, UCHIME algorithm for chimera removal, and DADA2 module for amplicon sequence variants (ASVs) denoising. Taxonomic annotation was conducted against the Silva database for bacteria and the UNITE database for fungi to obtain community abundance profiles. Each ASV feature table was rarefied to the read count of the sample with the minimum sequencing depth before α-diversity calculation. Some fine-tuned internal thresholds within the commercial cloud workflow were not manually configurable by the authors. The resulting ASV datasets were used to compare differences in microbial community structure among treatments.

### 2.4. Data Processing and Statistical Analysis

Raw data sorting was completed using Microsoft Excel 2019 (Microsoft, Washington, DC, USA). Statistical analysis was performed with SPSS 27.0 (IBM, New York, NY, USA) for one-way analysis of variance (ANOVA), and *p* < 0.05 was used to indicate significant differences between different treatment groups.

In addition, visual analysis of forest soil microbial communities was carried out on the Novogene Cloud Platform (https://magic.novogene.com), including abundance analysis, α/β diversity analysis, PICRUSt2 and FunGuild functional prediction. QIIME 2 software (version 202202) was used to generate species relative abundance diagrams and PCoA diagrams (vegan 2.7-3, Bray–Curtis method), and to calculate α-diversity indices (Shannon index, Simpson index, Pielou index, Chao1 index, Dominance index). To further explore the differences in community structure among grouped samples, LEfSe statistical analysis (microeco 2.0.0) was applied to test the significance of differences in species composition and community structure. PICRUSt2 (KO pathway) and FunGuild (mode guild) are bioinformatics software packages for predicting microbial functions based on marker genes (such as 16S rRNA and ITS). The core gene module of the KEGG database is the KEGG Orthology (KO) gene set, which is used to map bacterial and fungal gene sequences to corresponding functional profiles. By analyzing the correlation between amplicon annotation results and the corresponding functional database (KEGG), PICRUSt software (V2.5.1) and FunGuild were selected to predict and analyze the functions of microbial communities in ecological samples. ggNetView (0.1.0) was used to calculate the correlations between species to construct molecular network diagrams. Meanwhile, the psych 2.6.3 package was used to assess the correlation between phylum-level relative abundance and predicted functional profiles. Differential species and potential biomarkers were screened out through microeco 2.0.0 LEfSe statistical analysis, and Canoco 5.0 software (Microcomputer Power, New York, NY, USA) was used to perform redundancy analysis (RDA) on the aforementioned dominant microbial genera and environmental factors. Finally, the plspm 0.6.0 software package was used for partial least squares structural equation modeling (PLS-SEM) to clarify the direct and indirect effects of fire intensity, soil environmental variables, and the dynamics of microbial communities and functions.

## 3. Results and Analysis

### 3.1. Fire Severity Reshaped Soil Environmental Conditions

Our results (Table A3) showed that soil pH, nitrate nitrogen (NN), and easily oxidizable carbon (EOC) did not differ significantly among fire severity treatments (*p* > 0.05), whereas all other physicochemical variables exhibited significant differences (*p* < 0.05). Compared with the unburned control (U), fire-affected plots showed overall reductions in electrical conductivity (EC), available phosphorus (AP), and water-soluble organic carbon (WSOC).

AP exhibited a clear declining trend with increasing fire severity (Table A3): the low-severity treatment (L) showed a non-significant 9.17% decrease relative to U (*p* > 0.05), whereas moderate (M) and high (H) severity significantly reduced AP by 15.51% and 56.18%, respectively (*p* < 0.05). EC and WSOC followed a consistent pattern, with highest values in U, intermediate values in M, and lowest values in H. Ammonium nitrogen (AN) increased significantly under low severity (L), with a 24.10% increase relative to U (*p* < 0.05), but decreased substantially under moderate and high severity by 26.11% and 61.00%, respectively (*p* < 0.05).

Soil water content (SWC), available potassium (AK), and soil organic carbon (SOC) reached their highest values under moderate severity (Table A3). SWC was significantly higher in all burned treatments compared with U (*p* < 0.05). AK was significantly lower in L and H than in U, whereas SOC decreased significantly in L but increased significantly in M and H relative to the control (*p* < 0.05).

Soil aggregate stability indices, mean weight diameter (MWD) and geometric mean diameter (GMD), also peaked under moderate severity. MWD was lowest under high severity, whereas GMD was lowest under low severity; both indices were significantly higher in M compared with L and H (*p* < 0.05). Microbial biomass carbon (MBC), nitrogen (MBN), and phosphorus (MBP) increased progressively with fire severity, with significant differences among treatments (*p* < 0.05), except for MBP in L, which showed a non-significant increase. Relative to U, MBC increased by 40.39%, 54.98%, and 66.97% in L, M, and H, respectively; MBN increased by 14.20%, 43.82%, and 48.09%; and MBP increased by 0.94% (non-significant), 32.67%, and 39.61% (*p* < 0.05).

Overall, high-severity fire reduced the availability of key labile nutrients (AP and N) and labile organic carbon (WSOC), and weakened soil aggregate stability, whereas moderate fire enhanced soil moisture and helped maintain aggregate stability and organic carbon levels.

### 3.2. Fire Severity Drives Microbial Community Succession

The α-diversity of bacterial communities showed overall stability in response to fire severity (Table 1). No significant differences were observed among treatments for the Chao1 richness index, Pielou evenness index, or Shannon diversity index (*p* > 0.05). The Simpson index showed a decreasing trend with increasing fire severity, with significantly lower values in the H treatment compared with U (*p* < 0.05), whereas dominance exhibited the opposite pattern, being significantly higher in H than in U (*p* < 0.05).

In contrast, fungal α-diversity responded more sensitively to fire severity (Table 1), with all diversity indices showing significant differences among treatments (*p* < 0.05). In terms of richness, all burned treatments exhibited significantly lower Chao1 values than U (*p* < 0.05), with the lowest value observed in M (275.065 ± 5.391), which was significantly lower than both L and H (*p* < 0.05). Regarding evenness, Pielou’s index was significantly higher in all burned treatments compared with U (*p* < 0.05), although no significant differences were detected among fire severities. For overall diversity, L exhibited significantly higher Shannon and Simpson indices than all other treatments (*p* < 0.05), accompanied by significantly lower dominance; in contrast, M and H did not differ significantly from U for these indices (*p* > 0.05).

**Figure 2:**
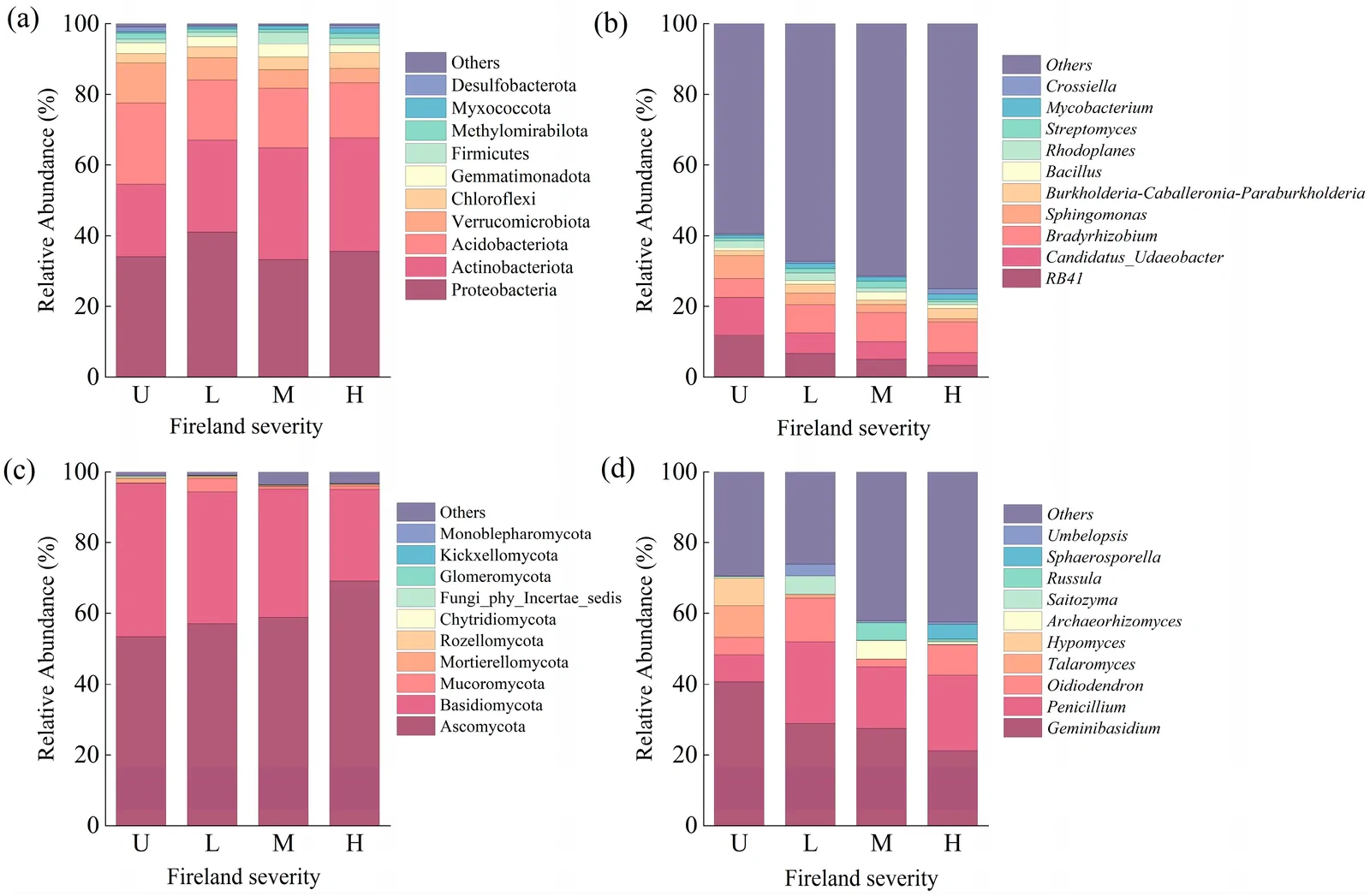
Shifts in bacterial and fungal community composition under different fire severities. Notes: Relative abundance of dominant microbial taxa across unburned (U), low (L), moderate (M), and high (H) fire severity treatments. (**a**) Bacterial community at the phylum level; (**b**) bacterial community at the genus level; (**c**) fungal community at the phylum level; and (**d**) fungal community at the genus level.

At the phylum level, bacterial community composition (Figure 2a,b) was dominated by Proteobacteria (33.25–41.02%), Actinobacteriota (20.59–32.12%), and Acidobacteriota (15.56–22.88%), which together accounted for more than 69% of total sequences. Verrucomicrobiota (4.15–11.40%), Chloroflexi (2.58–4.42%), and Gemmatimonadota (2.15–3.73%) represented the secondary dominant taxa. Proteobacteria reached its highest abundance in L, whereas Actinobacteriota increased progressively with fire severity, and both Acidobacteriota and Verrucomicrobiota declined. At the genus level, RB41 (3.29–11.89%) was the dominant bacterial taxon, followed by *Candidatus udaeobacter* (3.65–10.65%), *Bradyrhizobium* (5.26–8.26%), and *Sphingomonas* (0.86–6.55%). With increasing fire severity, RB41, *Candidatus udaeobacter*, and *Sphingomonas* decreased, whereas *Bradyrhizobium* increased.

Fungal communities (Figure 2c,d) were overwhelmingly dominated by Ascomycota (53.26–69.05%) and Basidiomycota (25.90–43.40%), together accounting for more than 90% of sequences. With increasing fire severity, Ascomycota increased gradually, whereas Basidiomycota declined. At the genus level, *Geminibasidium* (21.08–40.63%) was dominant, followed by *Penicillium* (7.62–23.03%), *Oidiodendron* (2.15–12.37%), and *Talaromyces* (0.10–8.98%). Increasing fire severity reduced the abundance of *Geminibasidium* (−19.55% in H vs. U) and *Talaromyces* (−8.88% in H vs. U), while significantly increasing *Penicillium* (+15.41% in H vs. U). Notably, *Hypomyces* was absent in all burned treatments, whereas *Archaeorhizomyces* and *Sphaerosporella* appeared exclusively in burned soils, with *Sphaerosporella* detected only under high severity.

**Figure A1:**
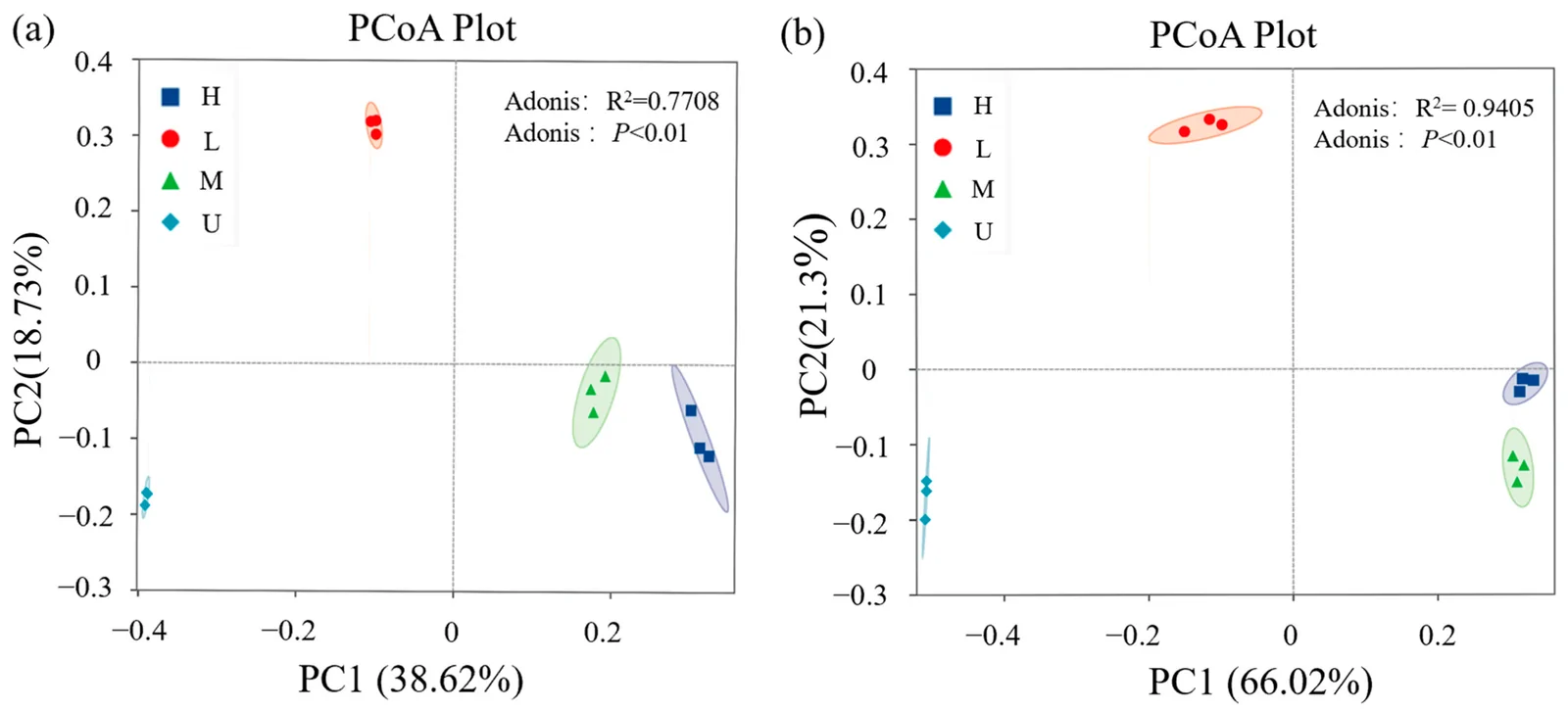
Fire severity drives distinct bacterial and fungal community differentiation. Notes: Principal coordinate analysis (PCoA) based on weighted UniFrac distances showing compositional differentiation of soil bacterial (**a**) and fungal (**b**) communities across fire-severity treatments. Each point represents an individual soil sample, and colors denote fire-severity treatments. The percentage values on the axes indicate the proportion of community variation explained by each principal coordinate. Differences in community structure among treatments were tested using Adonis analysis. U, unburned control; L, low-severity fire; M, moderate-severity fire; H, high-severity fire.

PCoA based on weighted UniFrac distances (Figure A1) showed that bacterial communities explained 38.62% and 18.73% of variation along PC1 and PC2, respectively (cumulative 57.35%) (Figure A1a), while fungal communities explained 66.02% and 21.30% (cumulative 87.32%) (Figure A1b). Samples clustered distinctly according to fire severity, indicating clear compositional divergence among treatments. Adonis analysis further confirmed that fire severity significantly affected both bacterial and fungal community structures (bacteria: R^2^ = 0.7708, *p* < 0.01; fungi: R^2^ = 0.9405, *p* < 0.01).

**Figure 3:**
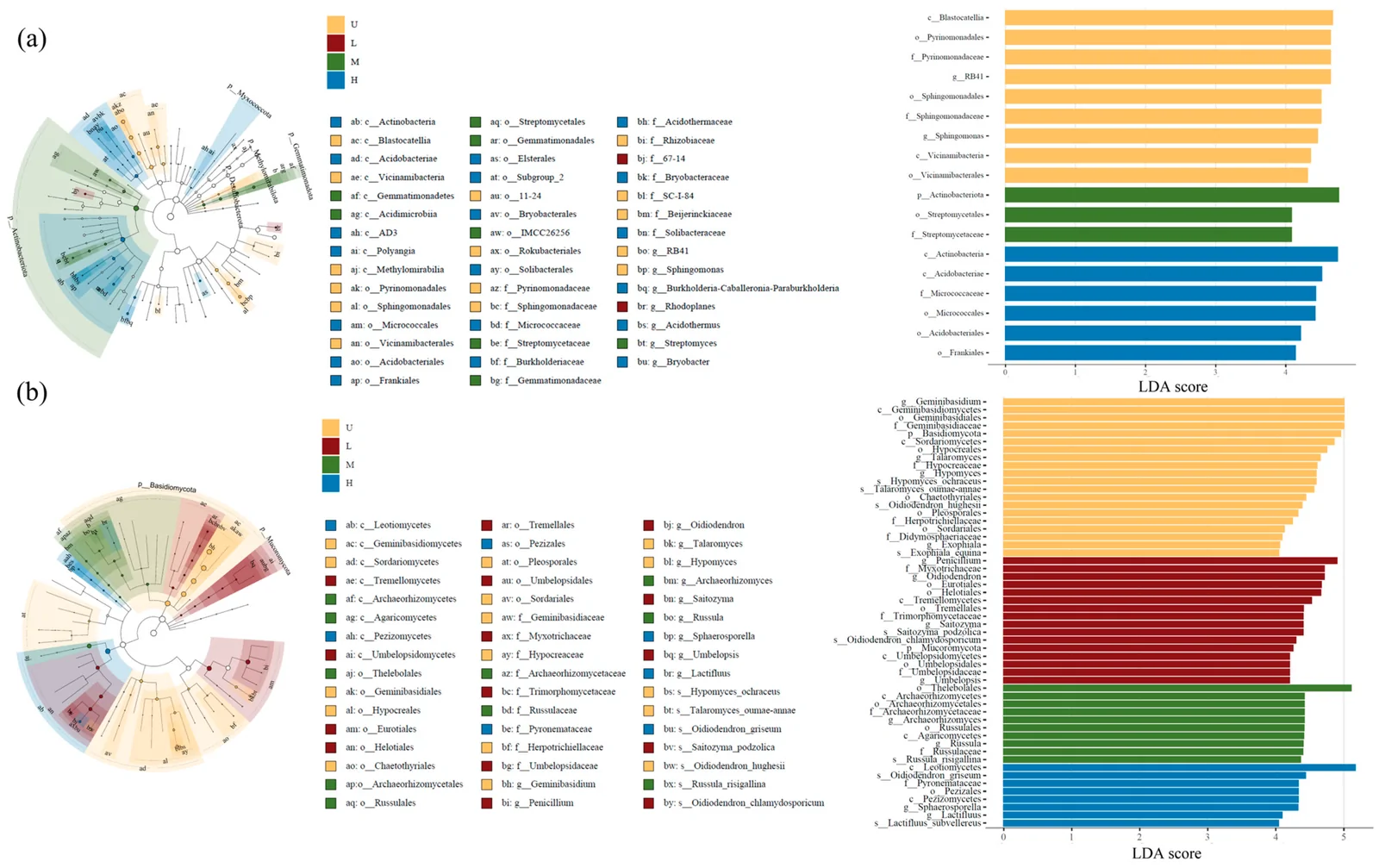
Differentially abundant microbial taxa across fire severity gradients identified by LEfSe analysis. Notes: Cladograms and LDA score bar plots showing differentially abundant bacterial (**a**) and fungal (**b**) taxa across unburned (U), low (L), moderate (M), and high (H) fire severity treatments. Taxa enriched in each treatment are indicated by different colors, and LDA scores represent the effect size of each biomarker.

LEfSe analysis identified key microbial biomarkers associated with different fire severities (Figure 3). In bacterial communities (Figure 3a), nine biomarkers were identified in U, including Blastocatellia and its affiliated taxa (Pyrinomonadales, Pyrinomonadaceae, and RB41), as well as *Sphingomonas* and related higher taxa. No significant biomarkers (LDA > 4) were detected in L, suggesting weak selective pressure under low-severity fire. In M, Actinobacteriota was strongly enriched, mainly driven by Streptomycetales and Streptomycetaceae. In H, dominant biomarkers included Actinobacteria, Micrococcales, Micrococcaceae, and Frankiales, along with enrichment of Acidobacteriia.

Fungal communities exhibited stronger and more distinct responses than bacteria, with clearly separated indicator taxa across treatments (Figure 3b). In U, 19 biomarkers were identified, dominated by *Geminibasidium* and its affiliated taxa, as well as *Talaromyces* and *Hypomyces*. In L, 16 taxa were enriched, including *Penicillium*, Eurotiales, and *Umbelopsis* (Mucoromycota). In M, Thelebolales, Archaeorhizomycetes, and *Russula* were significantly enriched. In H, only eight biomarkers were detected, with Leotiomycetes showing the highest LDA score; additionally, Pyronemataceae, Pezizomycetes, and *Sphaerosporella* were significantly enriched.

Overall, fire disturbance drove directional microbial succession. Bacterial communities exhibited relatively stable α-diversity and gradual compositional shifts, whereas fungal communities showed stronger and more persistent restructuring, with a progressive replacement of Basidiomycota by Ascomycota. Moderate fire severity emerged as a critical threshold shaping succession trajectories, beyond which fungal community divergence became more pronounced and indicator taxa more distinct.

### 3.3. Network Analysis and Functional Prediction

**Figure 4:**
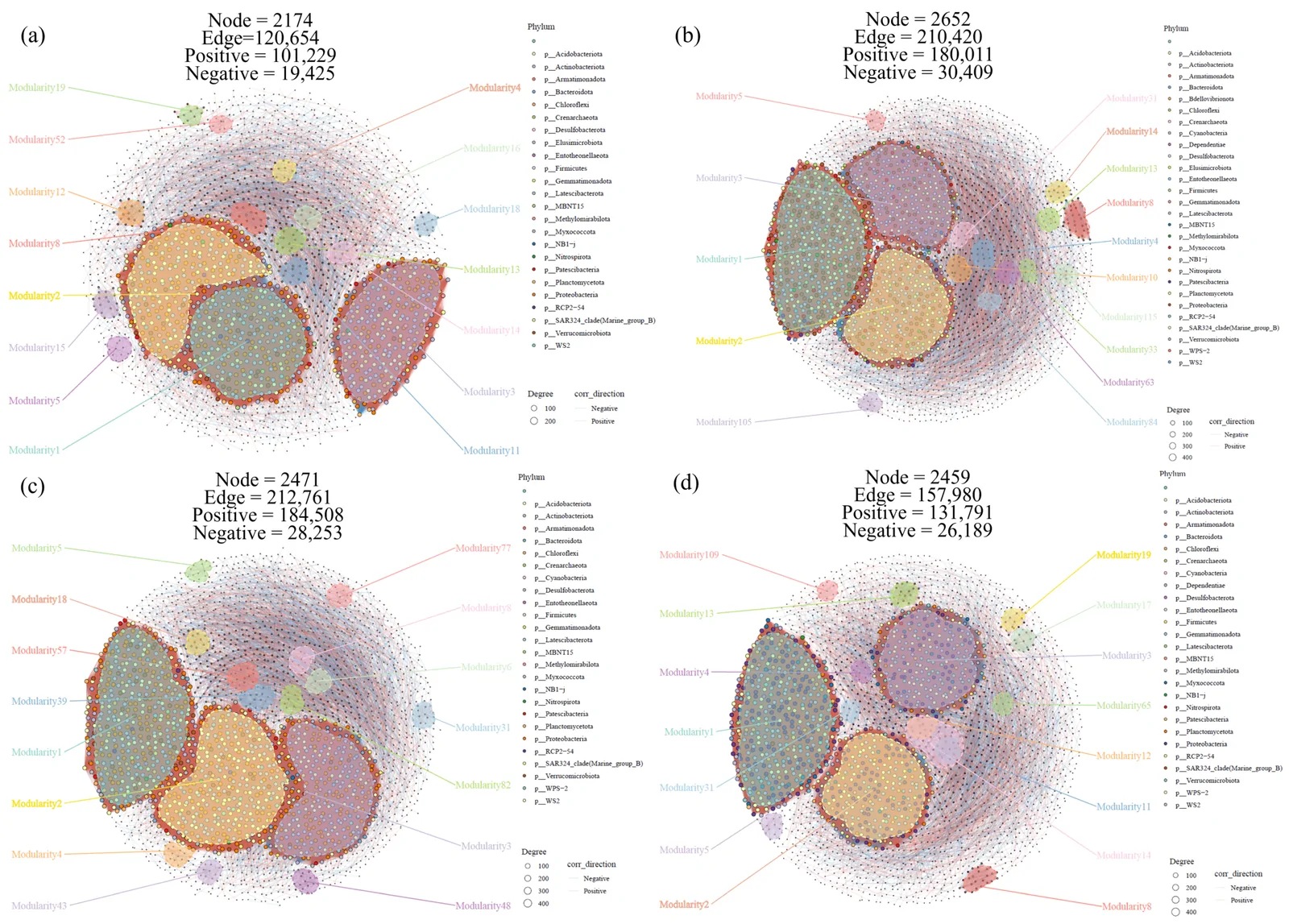
Bacterial co-occurrence networks across fire severity gradients. Co-occurrence networks of bacterial communities at the phylum level under different fire severity treatments in alpine forest soils. Notes: (**a**) unburned control (U); (**b**) low fire severity (L); (**c**) moderate fire severity (M); and (**d**) high fire severity (H). Nodes represent bacterial taxa, and edges represent significant correlations between taxa. Node size reflects connectivity, and different colors indicate modular structure within each network.

**Figure 5:**
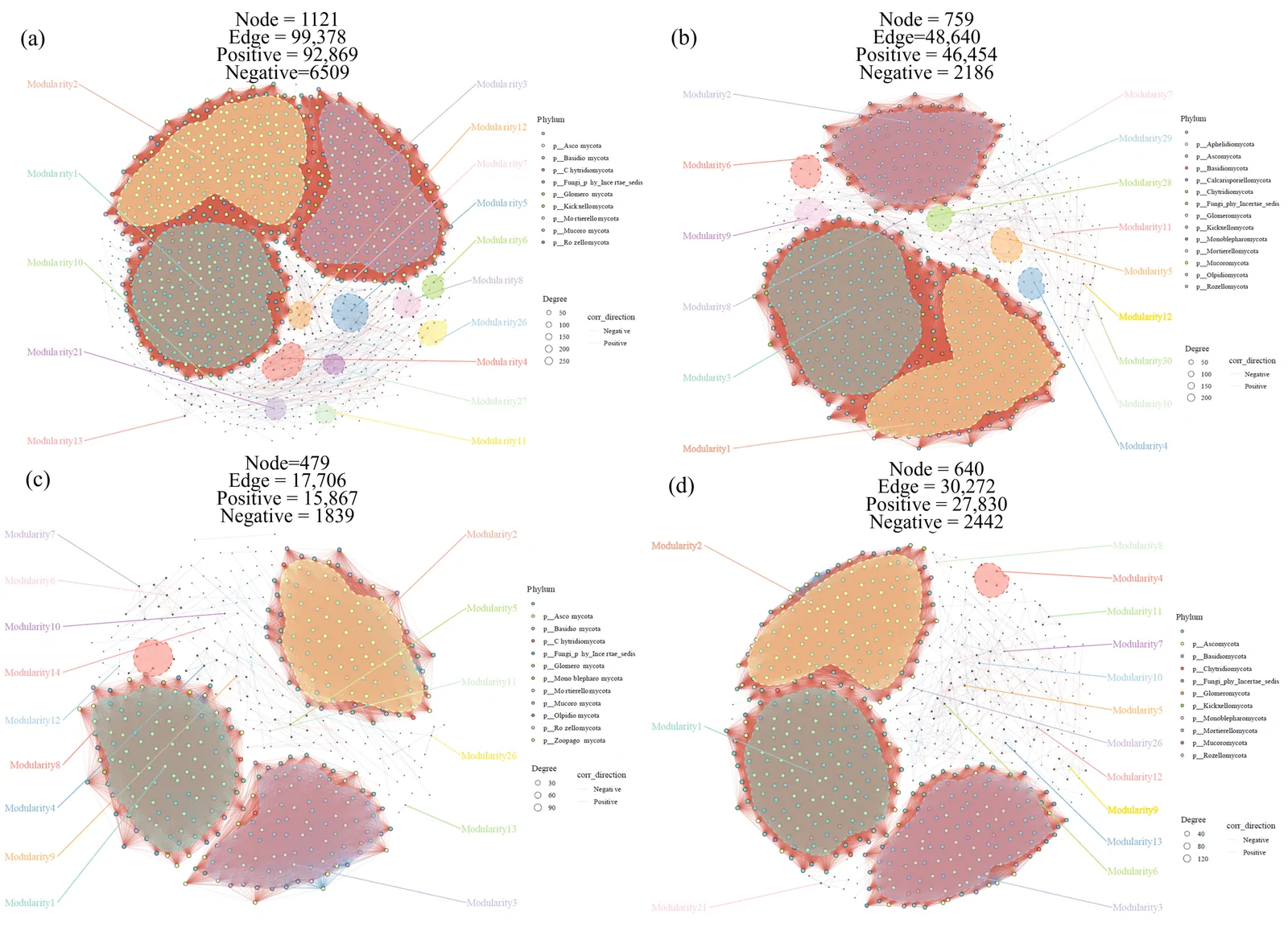
Fungal co-occurrence networks across fire severity gradients. Co-occurrence networks of fungal communities at the phylum level under different fire severity treatments in alpine forest soils. Notes: (**a**) unburned control (U); (**b**) low fire severity (L); (**c**) moderate fire severity (M); and (**d**) high fire severity (H). Nodes represent fungal taxa, and edges represent significant correlations among taxa. Node size reflects connectivity, and different colors indicate modular structure within each network. Differences in network topology among fire severity treatments reflect fire-induced shifts in fungal interaction patterns.

Bacterial networks were primarily composed of Acidobacteriota and Actinobacteriota (Figure 4). Under moderate severity, bacterial networks exhibited the highest number of edges, proportion of positive interactions, and complexity, followed by L and H, with U showing the lowest values. In contrast, fungal networks were dominated by Ascomycota and Basidiomycota (Figure 5). The unburned control exhibited the highest network complexity and connectivity, followed by L and H, whereas M showed the lowest values. Overall, bacterial networks were more complex than fungal networks, and moderate fire was associated with more connected networks. However, these patterns require confirmation in larger sample sizes.

**Figure 6:**
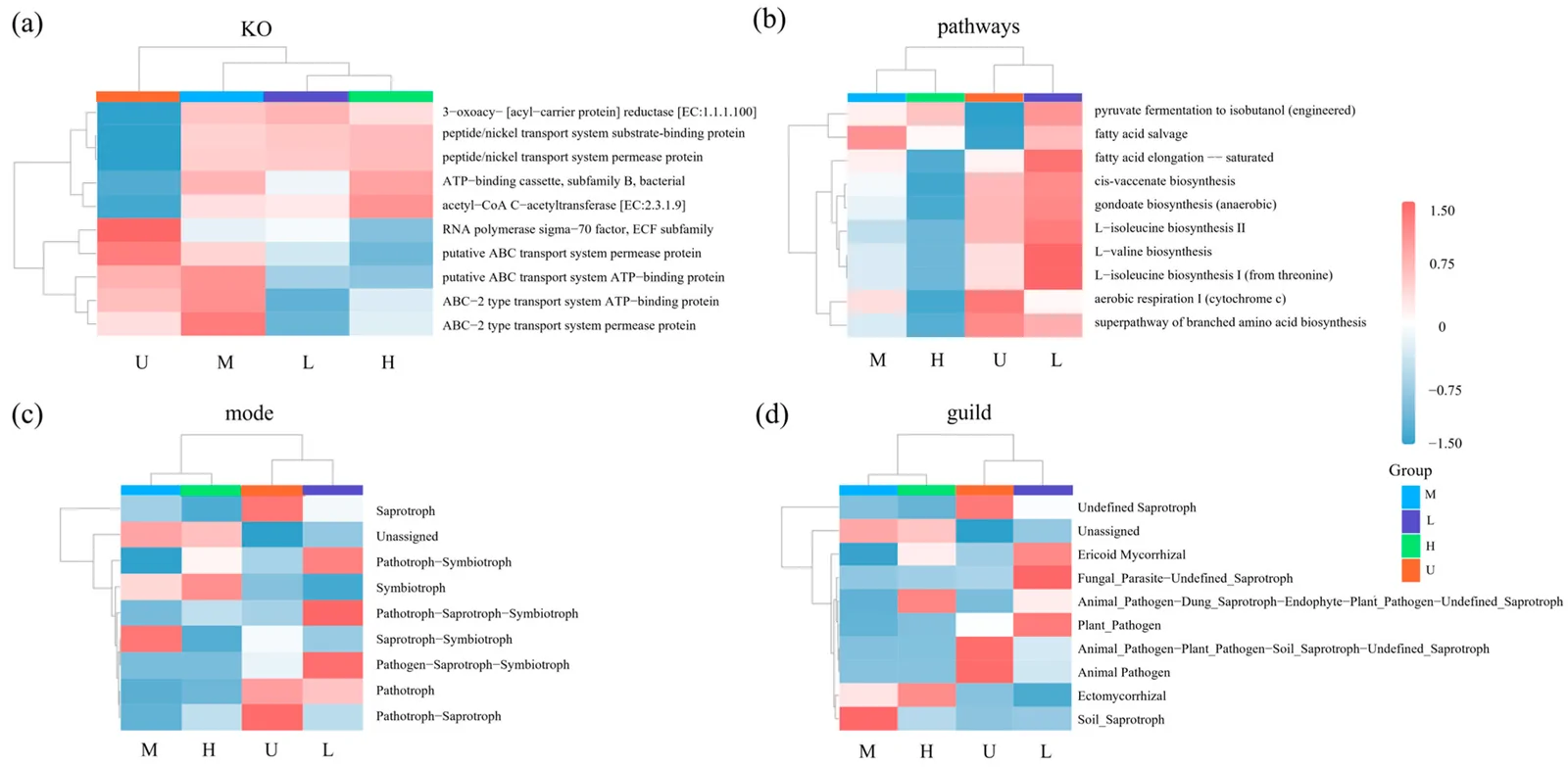
Predicted functional profiles of microbial communities across fire severity gradients. Notes: Heatmap of predicted microbial functional profiles across unburned (U), low (L), moderate (M), and high (H) fire severity treatments. (**a**) bacterial KEGG orthologs (KO), (**b**) bacterial metabolic pathways, (**c**) fungal trophic modes, and (**d**) fungal ecological guilds. Colors indicate normalized relative abundances of functional categories across treatments, with clustering reflecting similarities in functional composition. Functional profiles reveal clear separation among fire severity gradients, indicating fire-induced shifts in microbial metabolic strategies and fungal ecological lifestyles.

Functional prediction heatmaps (Figure 6) indicated clear separation between U and burned treatments at the bacterial KO level. Two major functional modules were identified. The first module, including peptide/nickel transport (ABC.PE.S, ABC.PE.P), acetyl-CoA metabolism (EC:2.3.1.9), and fatty acid biosynthesis (*fabG*), was lowest in U and significantly upregulated after fire, with *fabG* peaking in L and other functions peaking in H. The second module, including ABC transporters (ABC.CD.A, ABC.CD.P, ABC-2.A, ABC-2.P) and the stress regulator *rpoE*, showed mixed patterns, with *rpoE* and ABC.CD.P highest in U, ABC-2 subunits peaking in M, and generally reduced expression in L and H (Figure 6a). At the pathway level (Figure 6b), M and H clustered together, while U and L formed another group. Pyruvate fermentation to isobutanol and fatty acid salvage pathways were lowest in U and significantly increased after fire, peaking in L and M, respectively. Aerobic respiration, amino acid biosynthesis (L-valine, L-isoleucine), and fatty acid elongation pathways were lowest in H, while branched-chain amino acid synthesis and aerobic respiration I peaked in U.

Fungal functional modes and guilds showed clustering patterns similar to those of bacterial pathways (M + H vs. U + L). Saprotroph-dominated modes prevailed in U, whereas L was enriched in mixed trophic strategies. M and H showed increasing dominance of symbiotrophic and unassigned functions (Figure 6c). Guild-level analysis revealed that U was dominated by animal pathogens and undefined saprotrophs, whereas M was enriched in soil saprotrophs. H showed enrichment of ectomycorrhizal and animal pathogen–dung saprotroph guilds, while L was enriched in ericoid mycorrhizal fungi and plant pathogen-associated guilds (Figure 6d).

**Figure 7:**
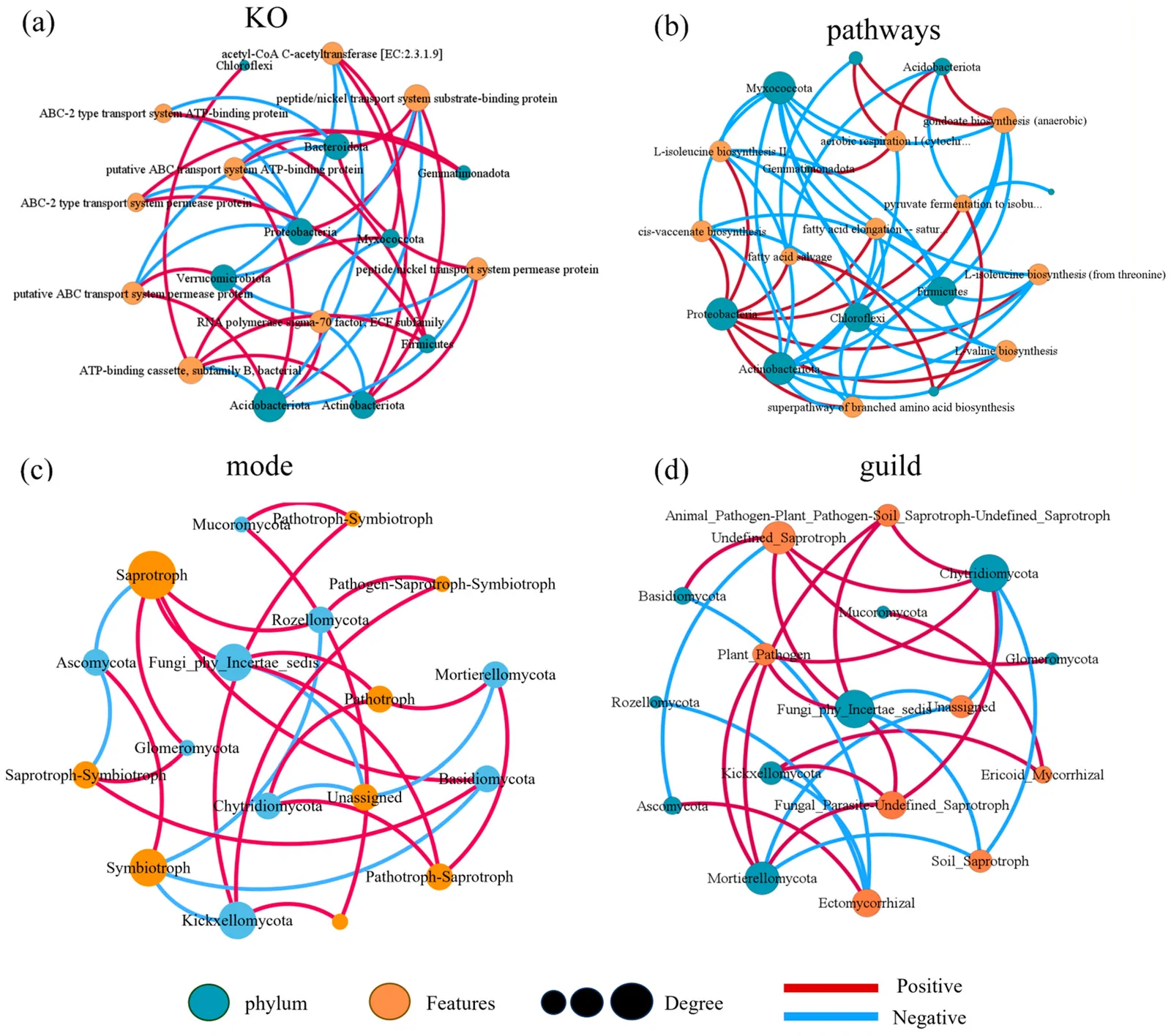
Coupling between microbial community composition and functional traits under fire disturbance. Notes: Network showing associations between microbial community composition and predicted functional traits across fire severity gradients in alpine forest soils. (**a**) bacterial phylum–KO associations, (**b**) bacterial phylum–pathway associations, (**c**) fungal phylum–trophic mode associations, and (**d**) fungal phylum–ecological guild associations. Nodes represent microbial taxa and functional categories, and edges represent significant positive (red) and negative (blue) associations. Node size reflects connectivity. These networks highlight coordinated shifts between microbial taxonomic composition and functional traits along fire severity gradients, indicating strong coupling between community structure and ecosystem functional potential.

Co-occurrence networks revealed fire-severity-dependent associations between microbial phyla and predicted functional traits (Figure 7). In the bacterial community, at the KEGG Orthology (KO) level (Figure 7a), Proteobacteria and Bacteroidota correlated positively with the peptide/nickel transport system (ABC.PE.S) and negatively with a subset of ABC transporters (ABC-2.A, ABC.CD.P/A). Firmicutes and Gemmatimonadetes displayed the opposite pattern, being positively associated with these transporters. Actinobacteria and Myxococcota showed positive correlations with acetyl-CoA C-acetyltransferase (EC 2.3.1.9) and the ABC transporter subfamily B (ABCB-BAC), and negative correlations with *rpoE*, whereas Acidobacteria and Verrucomicrobia exhibited the inverse pattern. At the pathway level (Figure 7b), Proteobacteria were positively associated with branched-chain amino acid biosynthesis, fatty acid elongation and fatty acid salvage pathways. Actinobacteria, Myxococcota, Chloroflexi and Firmicutes showed the opposite regulatory relationships and collectively displayed negative associations with gondoate biosynthesis.

In the fungal community, at the trophic mode level (Figure 7c), Ascomycota was exclusively positively correlated with Symbiotroph and negatively correlated with saprotroph. Basidiomycota and Glomeromycota exhibited the reverse pattern, correlating positively with saprotroph and negatively with Symbiotroph. Mortierellomycota, Chytridiomycota and unclassified fungi were positively associated with Pathotroph and the Pathotroph–Saprotroph composite. Mucoromycota and Kickxellomycota were positively associated with the Pathotroph–Symbiotroph composite and the triple trophic mode (Pathotroph–Saprotroph–Symbiotroph).

At the guild level (Figure 7d), undefined saprotroph correlated positively with Basidiomycota, Chytridiomycota and Glomeromycota, and negatively with Ascomycota. Ectomycorrhizal fungi correlated positively with Ascomycota and negatively with Basidiomycota, Rozellomycota and Kickxellomycota. Mortierellomycota, Chytridiomycota and unclassified fungi were positively correlated with plant pathogens, mycoparasite composites and multiple complex pathogen groups, and negatively correlated with soil saprotrophs. Ericoid mycorrhizal fungi were positively correlated exclusively with Mucoromycota and Kickxellomycota.

Overall, with increasing fire severity, microbial functional strategies shifted from resource acquisition towards stress adaptation and saprotrophic or symbiotic interactions. Fungi were generally more fire-sensitive, whereas bacteria enhanced their adaptability through metabolic reorganization.

### 3.4. Regulation of Microbial Responses by Soil Environmental Factors

**Figure A2:**
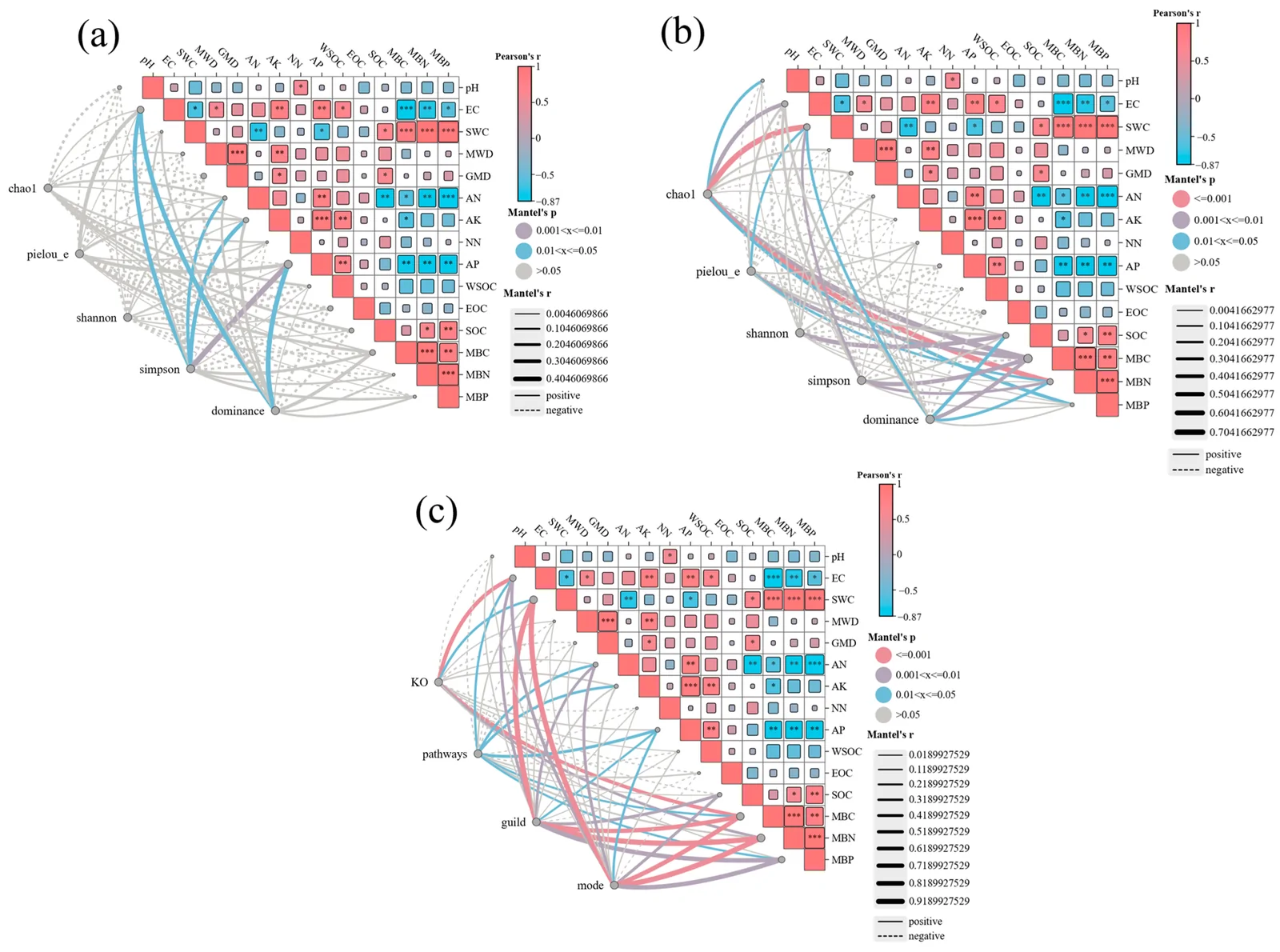
Relationships between microbial diversity, microbial function and post-fire soil environmental factors. Notes: Pearson correlation analysis and Mantel tests showing associations between soil physicochemical properties, microbial biomass and microbial diversity or functional profiles. Relationships are shown for bacterial alpha diversity (**a**), fungal alpha diversity (**b**) and microbial functional profiles (**c**). Pearson correlations among soil variables are shown as colored cells, while Mantel correlations are represented by connecting lines. Line width indicates Mantel’s r value, and line color indicates statistical significance. * *p* < 0.05, ** *p* < 0.01, *** *p* < 0.001. EC, electrical conductivity; SWC, soil water content; SOC, soil organic carbon; AN, ammonium nitrogen; AP, available phosphorus; AK, available potassium; MBC, microbial biomass carbon; MBN, microbial biomass nitrogen; MBP, microbial biomass phosphorus.

Mantel tests (Figure A2) revealed significant associations between microbial indices and soil physicochemical properties. In bacterial communities (Figure A2a), the Simpson index was positively correlated with AP (*p* < 0.01) and positively correlated with EC, AN, and AK (*p* < 0.05). Dominance was positively associated with EC and AP (*p* < 0.05). In fungal communities (Figure A2b), Chao1 richness showed strong positive correlations with EC, MBC, SWC, and MBN (*p* < 0.01), and significant correlations with pH and MBP (*p* < 0.05). Pielou evenness was strongly correlated with MBC (*p* < 0.01) and significantly correlated with MBP and SWC (*p* < 0.05). Shannon diversity was strongly associated with SOC (*p* < 0.01). The Simpson index was strongly correlated with SWC, MBC, and MBN (*p* < 0.01), while dominance was strongly associated with MBC and significantly correlated with SWC, SOC, and MBN (*p* < 0.05). For functional traits (Figure A2c), KO functions were strongly correlated with EC, MBC, and MBN (*p* < 0.01), and significantly correlated with SWC (*p* < 0.05). Pathway-level functions were significantly correlated with EC, AN, AK, AP, MBC, and MBP (*p* < 0.05). Fungal mode and guild structures showed consistent patterns, both being strongly correlated with EC, SWC, AN, SOC, MBC, MBN, and MBP (*p* < 0.01).

**Figure 8:**
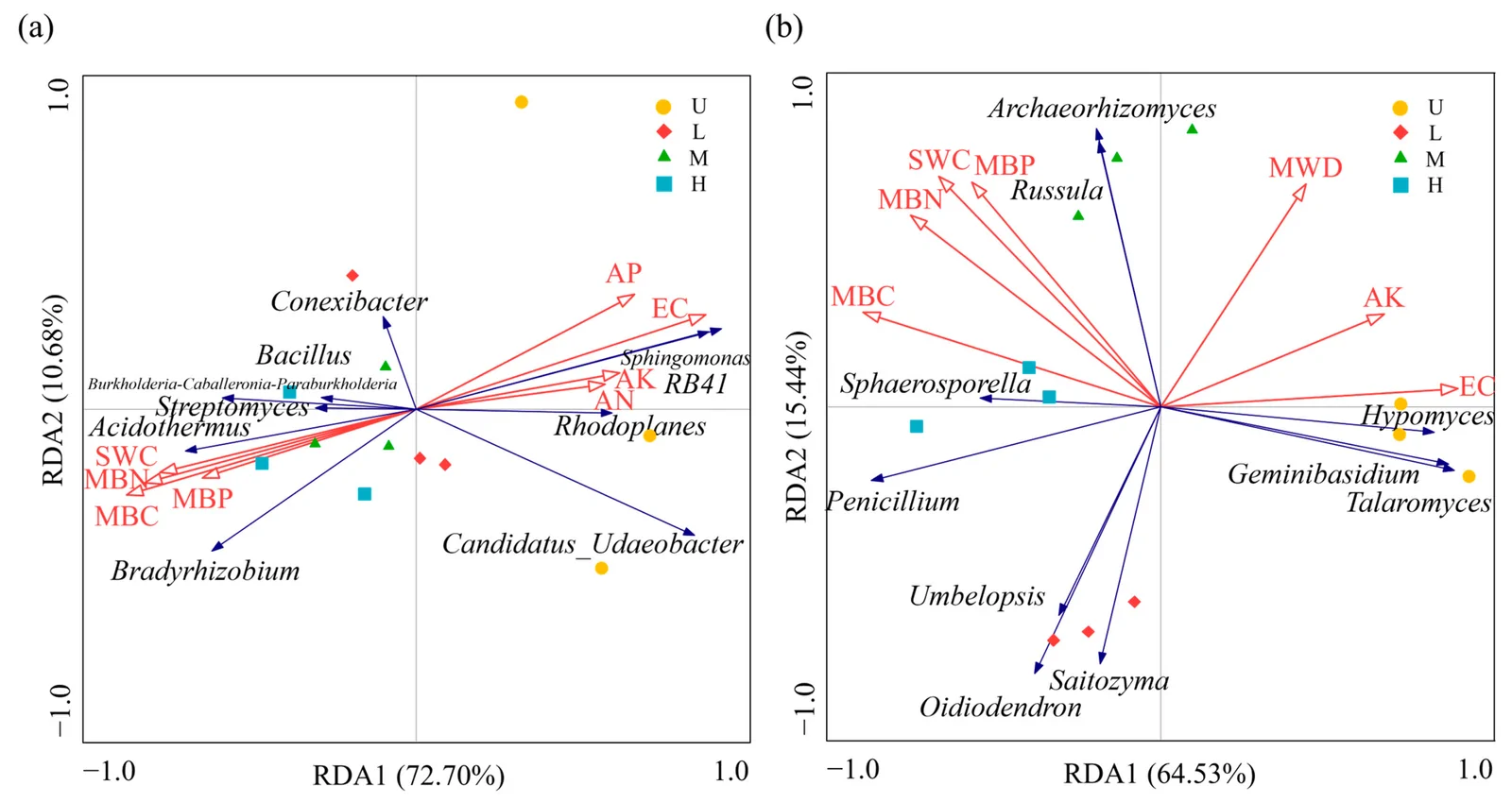
Redundancy analysis of microbial communities and soil properties. Notes: RDA showing relationships between dominant bacterial (**a**) and fungal (**b**) genera and soil physicochemical properties across fire severity treatments. Arrows represent environmental variables and points represent. Red arrows represent environmental variables (SWC, MBC, MBN, MBP, AP, AK, AN, EC, MWD); Blue arrows represent dominant microbial genera; their direction and length indicate the correlation with the sorting axis; scatter points represent soil samples, with yellow circles, red diamonds, green triangles, and blue squares denoting unburned controls (U), low-severity (L), moderate-severity (M), and high-severity (H) samples, respectively.

Redundancy analysis (RDA) further supported these patterns. For bacteria (Figure 8a), RDA1 and RDA2 explained 72.70% and 10.68% of the variance, respectively (cumulative 83.38%). AN was the most influential factor (25.4%, *p* = 0.044), followed by SWC (22.2%), MBC (14.0%), and MBP (10.7%) (Table A4). For fungi (Figure 8b), RDA1 and RDA2 explained 64.53% and 15.44% of the variance (cumulative 79.97%). MBN was the strongest driver (23.8%, *p* = 0.016), followed by AK (22.6%), MWD (20.7%), and MBC (11.0%).

**Figure 9:**
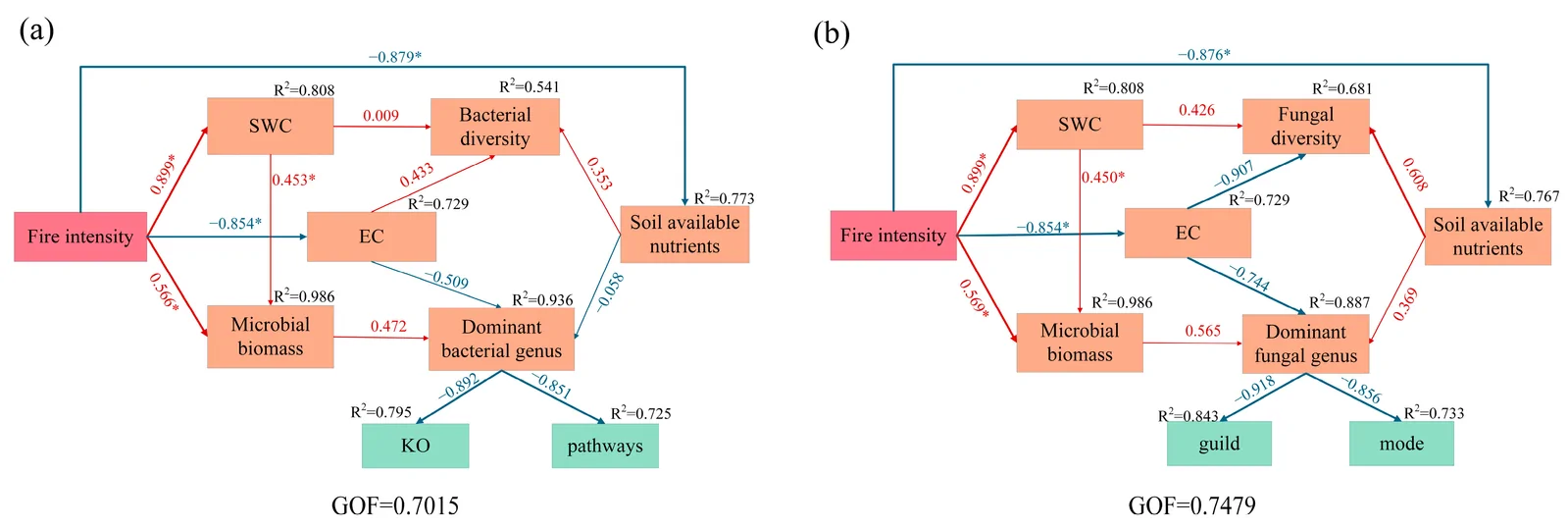
Structural equation modeling of fire severity, soil properties, and microbial responses. Notes: Partial least squares structural equation modeling (PLS-SEM) showing the effects of fire severity on soil properties, microbial biomass, community structure, and functional traits in bacteria (**a**) and fungi (**b**). Numbers on arrows indicate standardized path coefficients; red and blue arrows represent positive and negative effects, respectively. R^2^ indicates explained variance of each response variable, and GOF represents model fit. * indicates that the load coefficient is significant at the 0.05 level.

Partial least squares structural equation modeling (PLS-SEM) (Figure 9) showed good model fits (GOF = 0.7015 for bacteria and 0.7479 for fungi). Fire severity exerted strong negative effects on EC and available nutrients (−0.854 and −0.879 in bacteria; −0.854 and −0.876 in fungi; *p* < 0.05), while positively affecting SWC and microbial biomass (0.899 and 0.566/0.569; *p* < 0.05). SWC positively influenced microbial biomass (0.453 in bacteria; 0.450 in fungi) and fungal diversity (0.426), but had minimal effect on bacterial diversity (0.009). Microbial biomass positively regulated dominant bacterial (0.472) and fungal genera (0.565), which in turn negatively regulated functional genes and pathways in bacteria (−0.892, −0.851) and fungal trophic modes and guilds (−0.918, −0.856), respectively.

## 4. Discussion

### 4.1. Long-Term Legacy Effects of Fire Severity on Belowground Environments

Forest fires not only restructure aboveground vegetation and ecosystem architecture but also generate long-lasting ecological legacy effects through alterations in soil resource availability and microenvironmental conditions, thereby continuously shaping post-fire recovery trajectories [26]. In the present study, we found that eight years after fire disturbance, significant differences in soil organic carbon (SOC), soil water content (SWC), available nitrogen (AN), available phosphorus (AP), and microbial biomass (MBC and MBN) persisted among fire severity treatments. These results indicate that fire-induced heterogeneity in belowground environments does not dissipate over time but instead continues to regulate ecosystem recovery processes [2,13].

Distinct fire severities generated markedly divergent recovery trajectories. In this study, moderate-severity fire (M) exhibited the highest SOC, SWC, and aggregate stability, suggesting a transition from an early-stage resource depletion phase to a later-stage resource accumulation phase after eight years of recovery. This shift is likely attributable to enhanced litter inputs from post-fire vegetation recovery, increased root-derived carbon inputs, and improved soil aggregation that enhances water retention and provides physical protection for organic matter [27,28]. In contrast, although SOC and SWC partially recovered under high-severity fire (H), AN and AP remained significantly lower than in other treatments, indicating persistent nutrient limitation. Fire-induced volatilization of organic nitrogen and the conversion of phosphorus into less available forms, combined with delayed vegetation and root recovery in high-severity areas, likely constrained nutrient replenishment efficiency [29]. Consequently, despite partial recovery of carbon pools, high-severity burned soils may remain in a long-term resource-limited state.

Notably, although AN and AP were lowest under high-severity fire, MBC and MBN were still significantly higher than in the unburned control. This pattern likely reflects the combined effects of increased SWC and partially recovered SOC, which alleviate moisture stress and provide carbon substrates, together with microbial community shifts toward stress-tolerant and oligotrophic-adapted taxa that sustain relatively high microbial biomass. In the moderate-severity treatment, the concurrent advantages of higher moisture availability, carbon inputs, and aggregate stability further supported elevated microbial biomass.

The alpine forests of western Sichuan are characterized by low temperatures, slow organic matter decomposition, and limited nutrient mineralization rates, as well as short growing seasons. As a result, pronounced differences in belowground environmental conditions can persist even eight years after fire disturbance [30]. In addition, large amounts of pyrogenic carbon formed after fire decompose slowly under cold conditions, further reinforcing long-term legacy effects in soil systems [31]. Collectively, these factors indicate that alpine forest recovery is particularly sensitive to fire severity and is governed by persistent ecological memory effects mediated through long-lasting alterations in water, carbon, and nutrient availability.

### 4.2. Environmental Filtering Drives Microbial Community Succession

Fire severity does not directly determine bacterial community composition; rather, it indirectly shapes microbial succession by modifying soil environmental conditions such as water and nutrient availability [32]. In the present study, redundancy analysis (RDA), Mantel tests, and partial least squares structural equation modeling (PLS-SEM) consistently demonstrated that EC, SWC, SOC, AN, MBC, and MBN were more strongly associated with microbial community variation than fire severity per se, supporting this interpretation.

Under high-severity fire, Actinobacteriota was significantly enriched, whereas RB41, *Sphingomonas*, and *Candidatus udaeobacter* declined markedly. This pattern likely reflects the strong stress tolerance and thermoresistance of Actinobacteriota, which enables utilization of pyrogenic organic matter as carbon sources in nutrient-poor environments [33]. In contrast, RB41 (within Acidobacteriota) is typically associated with stable, acidic, and oligotrophic environments [34]. RDA results further showed that these declining taxa were positively correlated with available nutrients (AN, AP, AK), which were substantially reduced under high fire severity, thereby constraining their abundance [33]. In addition, the significant increase in *Bradyrhizobium* under high-severity fire likely reflects nitrogen limitation induced by vegetation mortality and subsequent selection for biological nitrogen fixation as a compensatory mechanism.

Notably, bacterial network complexity peaked under moderate fire severity (M), which can be attributed to relatively high SWC, SOC, and MBC in this treatment. Moderate fire generally does not completely destroy soil structure; instead, ash inputs and partial carbonization of litter can enhance water retention and resource heterogeneity, thereby promoting microbial coexistence and interaction complexity [35,36,37].

Fungal communities exhibited stronger sensitivity to fire disturbance than bacteria. With increasing fire severity, the relative abundance of Ascomycota increased continuously, whereas *Basidiomycota* declined. Ascomycetes are widely recognized as pioneer taxa in post-fire environments due to their rapid colonization capacity [12], whereas basidiomycetes are more dependent on stable host plants and intact mycorrhizal systems, making them more vulnerable to fire disturbance [38].

At finer taxonomic resolution, *Penicillium* increased significantly under high fire severity, while *Geminibasidium* and *Talaromyces* declined. This shift is closely associated with the increased availability of pyrogenic residues and labile organic substrates following fire. *Penicillium* is a well-known post-fire fungal taxon [39], with genomic capacities for aromatic compound degradation, enabling efficient decomposition of lignin and thermally altered carbon compounds [40]. Continued litter inputs during vegetation recovery, together with persistent pyrogenic substrates, provide diverse resources that favor *Penicillium* proliferation [41]. In contrast, early-stage pioneer fungi such as *Geminibasidium* preferentially utilize labile organic matter [42], consistent with its positive correlation with available potassium (AK) observed in RDA and structural equation modeling results. Ecological succession patterns therefore reflect niche differentiation, where *Geminibasidium* dominates early stages, whereas *Penicillium* gradually becomes dominant in later stages due to its ability to decompose more complex substrates such as lignin and charred organic matter. This transition explains the shift from symbiosis-dominated fungal communities toward decomposition-oriented assemblages under high fire severity.

Importantly, *Sphaerosporella* was detected exclusively in high-severity plots, consistent with its classification as a pyrophilous fungus capable of rapid colonization of fire-affected substrates [43], indicating the formation of specialized post-fire ecological niches. In contrast, moderate-severity fire promoted enrichment of *Russula* and *Archaeorhizomycetes*, where *Russula* represents a typical ectomycorrhizal fungus whose recovery suggests partial preservation and early reassembly of mycorrhizal networks [44].

Overall, fungal Chao1 richness decreased significantly across all burned treatments, and fungal networks were most complex in unburned soils, indicating that fungi are more sensitive to fire disturbance than bacteria. Their hyphal networks and plant-associated interactions had not fully recovered even eight years post-fire, consistent with previous findings that fungal communities exhibit higher disturbance sensitivity and are strongly reshaped by pyrophilous taxa following fire events [10]. Collectively, soil moisture and nutrient availability act as dominant ecological filters, driving microbial communities toward distinct survival strategies and shaping long-term post-fire successional trajectories.

### 4.3. Fire Severity Drives Microbial Functional Shifts from Resource Acquisition to Stress Adaptation

Predicted changes in microbial functional traits provide complementary insight into belowground ecosystem reconstruction beyond taxonomic composition alone. These functional profiles were inferred from taxonomic composition and therefore represent predicted functional potential, not direct evidence of in situ gene expression, enzyme activity, or metabolite flux. Across fire severity gradients, predicted bacterial metabolic pathways and fungal trophic modes showed significant divergence and strong correlations with SOC, SWC, MBC, MBN, and EC, suggesting that functional potential is closely associated with shifts in soil resource availability. Under low-severity fire, increased nutrient availability (AN and AP) and relatively low environmental stress were associated with predicted enrichment of aerobic respiration and amino acid and fatty acid biosynthesis pathways in bacteria, consistent with a resource-acquisition strategy. Members of Proteobacteria were positively associated with these predicted core metabolic functions, consistent with a copiotrophic lifestyle strategy [45].

Under moderate-severity fire, oxidative stress may have been associated with membrane lipid peroxidation without necessarily causing complete microbial mortality, potentially leading to free fatty acid accumulation in the soil. Predicted enrichment of fatty acid salvage pathways is consistent with potential membrane repair while conserving energy [46]. Concurrently, predicted enrichment of ABC transporters may reflect enhanced material transport requirements and metabolic reallocation toward biosynthetic processes, consistent with a potential recovery-oriented strategy. Under high-severity fire, severe nutrient depletion (AN, AP, AK), complex carbon substrates, and increased environmental variability were associated with predicted enrichment of ABC transport systems, lipid metabolism, and acetyl-CoA-related pathways, together with lower predicted abundance of core metabolic functions. This pattern may suggest the synthesis of stress-resistant membrane lipids, energy storage strategies, and maintenance of redox balance, including predicted pyruvate-to-isobutanol-related pathways for excess reducing power dissipation. The enrichment of Actinobacteria, strongly correlated with these predicted functions, is consistent with a transition toward stress tolerance and survival-oriented strategies [47]. Predicted fungal functional shifts were closely related to potential changes in carbon cycling pathways following fire. In unburned soils, predicted pathogenic fungi were relatively enriched, which may reflect a balanced antagonistic network structure rather than ecosystem degradation. Fungal co-occurrence networks in unburned soils exhibited the highest complexity, suggesting more interconnected fungal associations. Low-severity fire may have moderately altered microhabitats (e.g., pH and moisture), potentially disrupting competitive equilibria and favoring fungi with broad trophic strategies, including saprotroph–pathogen combinations [48], consistent with previous findings that low-intensity fire primarily restructures microbial interactions rather than community composition [4]. Under moderate- and high-severity fire, predicted pathogenic fungi were suppressed, whereas predicted mycorrhizal fungi (ectomycorrhizal and ericoid mycorrhizal taxa) and saprotrophic fungi became increasingly dominant. High-severity fire may have eliminated heat-sensitive pathogens [6], while subsequent plant recolonization may have facilitated recruitment of ericoid mycorrhizal fungi through root exudates, potentially promoting the re-establishment of plant–microbe symbioses [49]. Simultaneously, abundant dead woody debris may have provided substantial lignocellulosic substrates, potentially supporting saprotrophic fungi capable of degrading cellulose and lignin. Pyrophilous saprotrophs with thick-walled spores may have rapidly colonized post-fire environments, consistent with a shift from plant-associated to detritus-driven fungal communities [10,11]. This transition was particularly pronounced under moderate fire (potential mycorrhizal recovery) and high fire (potential saprotroph dominance and stress adaptation). The increased abundance of unassigned taxa under high severity suggests incomplete functional recovery and persistent instability of fungal functional systems, likely influenced by alpine environmental constraints and prolonged recovery times [50,51].

Overall, predicted microbial life-history strategies shifted along a fire severity gradient: from resource acquisition (low severity), to recovery and restructuring (moderate severity), to stress tolerance and survival (high severity), suggesting a potential coupling between soil resource dynamics, vegetation recovery, and microbial functional potential. While these functional inferences are based on taxonomic prediction and therefore represent functional potential rather than in situ activity, they provide a coherent framework and testable hypotheses linking fire severity to belowground ecosystem reassembly. Future studies combining metatranscriptomics, enzyme activity assays, and targeted metabolomics are needed to test whether the predicted pathways are actually active in soil.

### 4.4. Implications for Ecosystem Resilience and Post-Fire Management

Under global climate change, increasing frequency of extreme heat and drought events is driving a rise in wildfire severity [52]. High-severity fire has thus become a major ecological threat, affecting not only aboveground vegetation but also inducing persistent alterations in belowground processes [53]. In this study, significant differences in soil properties, microbial community structure, and functional traits persisted eight years after fire, with fungal richness significantly reduced across all burned treatments and network complexity highest in unburned soils. These findings confirm that fungi are more sensitive to fire than bacteria, with incomplete recovery of hyphal networks and plant-associated interactions [10].

High-severity fire substantially suppressed available nutrients and mycorrhizal recovery relative to moderate fire, despite partial recovery of SOC and SWC, indicating pronounced asynchronous recovery, where belowground nutrient cycling lags behind aboveground vegetation recovery [13]. Therefore, assessments based solely on vegetation recovery may underestimate belowground functional impairment. Mycorrhizal fungi play a critical role in plant establishment and community assembly [54]. Their suppression under high-severity fire, together with increased proportions of pathogenic and opportunistic saprotrophic fungi, suggests incomplete reconstruction of plant–microbe symbioses. This functional imbalance may reduce seedling establishment and plant stress resistance, thereby delaying forest recovery [55].

Accordingly, high-severity fire induces long-term feedbacks between aboveground vegetation loss and belowground microbial functional degradation, forming a coupled feedback loop that may underlie slow recovery in alpine forests. In contrast, moderate-severity fire enhanced SOC, SWC, and bacterial network complexity, supporting ecosystem resilience theory, which proposes that intermediate disturbance can enhance system stability through resource redistribution and niche restructuring [56]. High-severity fire, however, exceeds ecological resilience thresholds, resulting in long-term deviation from pre-disturbance conditions that cannot be rapidly restored through natural succession alone.

From a management perspective, fire severity is likely a greater ecological risk factor than fire frequency under future climate scenarios, as it more readily exceeds recovery thresholds and induces persistent functional degradation. In low- and moderate-severity burned areas, natural recovery may be sufficient to maintain belowground processes. In contrast, high-severity areas require active intervention, including enhancement of soil organic matter, improvement of water retention capacity, and restoration of mycorrhizal networks (e.g., retention of coarse woody debris, organic amendments, and mycorrhizal inoculation) [57,58]. Given the slow recovery dynamics of alpine ecosystems, long-term monitoring combined with adaptive management strategies is essential.

## 5. Conclusions

This study shows that, eight years after fire disturbance, soil microbial community composition and functional attributes in alpine forests of western Sichuan remain strongly differentiated along fire severity gradients, highlighting persistent legacy effects of fire on belowground ecosystems. Fire severity influences microbial community assembly and functional potential primarily through long-term alterations in soil organic carbon, moisture availability, available nitrogen and phosphorus, and microbial biomass.

Under high-severity fire, stress-tolerant and saprotrophic taxa (e.g., Actinobacteriota) were significantly enriched, whereas oligotrophic bacteria (e.g., RB41) and basidiomycete mycorrhizal fungi declined, suggesting a shift from relatively stable plant–microbe associations toward communities with greater representation of decomposer-associated taxa. Meanwhile, increased representation of ABC transport activity, lipid metabolism, and saprotrophic functions, together with reduced core metabolic functions, indicates a potential transition from resource-acquisition strategies toward stress-response strategies under severe fire conditions. Moderate-severity fire maintained higher soil moisture and organic carbon levels, which were associated with increased resource heterogeneity and microbial network connectivity, as well as higher abundances of specific mycorrhizal taxa such as *Russula*. Low-severity fire maintained relatively stable microbial functional profiles, including pathways related to branched-chain amino acid biosynthesis. Overall, fungal communities exhibited greater sensitivity to fire severity than bacterial communities, characterized by reduced richness, altered network properties, and increased proportions of unclassified functional groups, suggesting a slower recovery trajectory following fire disturbance.

Given the economic and logistical constraints associated with restoring remote alpine forests, post-fire management should prioritize ecosystem protection and natural recovery. These forests provide important ecosystem services, including water regulation, soil stabilization, carbon storage, and biodiversity conservation. We recommend: (i) strengthening fire prevention and minimizing further degradation in high-severity burned areas; (ii) promoting natural recovery with long-term monitoring in moderate-severity areas; and (iii) implementing site-specific, cost-effective interventions (e.g., mycorrhizal inoculation and organic amendments) only where ecological benefits justify their application. Adaptive and long-term management approaches are essential for supporting post-fire ecosystem recovery.

Because alpine forest ecosystems are characterized by slow biogeochemical turnover and prolonged recovery processes, post-fire ecological recovery may occur over decadal timescales. However, the present study focused on the 0–10 cm topsoil layer, and whether these legacy effects extend to deeper soil horizons remains unresolved. Future studies should incorporate deeper soil profiles, quantitative vegetation assessments, and path-based analyses to better disentangle the direct and indirect effects of fire severity on belowground recovery. Field-based restoration experiments will also be valuable for evaluating the effectiveness and feasibility of different recovery approaches, particularly in high-severity burned areas.

## Figures and Tables

**Table 1:** Microbial α-diversity responses to fire severity gradients.

Group	Fire Severity	Chao1	Pielou’s Evenness	Shannon Index	Simpson Index	Dominance
Bacteria	U	1511.868 ± 30.595 b	0.910 ± 0.018 a	9.611 ± 0.171 a	0.997 ± 0.001 a	0.003 ± 0.001 d
	L	1772.675 ± 177.896 a	0.900 ± 0.006 a	9.708 ± 0.176 a	0.996 ± 0.001 ab	0.004 ± 0.001 ab
	M	1607.052 ± 73.911 ab	0.904 ± 0.010 a	9.622 ± 0.163 a	0.996 ± 0.002 ab	0.004 ± 0.002 ab
	H	1691.779 ± 135.576 ab	0.888 ± 0.015 a	9.525 ± 0.176 a	0.994 ± 0.002 b	0.006 ± 0.002 a
Fungi	U	553.278 ± 10.906 a	0.518 ± 0.030 c	4.720 ± 0.267 b	0.853 ± 0.042 b	0.147 ± 0.042 a
	L	392.333 ± 35.781 b	0.608 ± 0.019 a	5.235 ± 0.229 a	0.941 ± 0.008 a	0.059 ± 0.008 b
	M	275.309 ± 8.939 d	0.568 ± 0.011 b	4.599 ± 0.089 b	0.906 ± 0.004 a	0.094 ± 0.004 b
	H	348.486 ± 12.604 c	0.574 ± 0.017 ab	4.842 ± 0.113 b	0.917 ± 0.011 a	0.083 ± 0.011 b

Notes: Alpha-diversity metrics of bacterial and fungal communities across fire severity treatments. Different lowercase letters denote significant differences among treatments (*p* < 0.05).

## Data Availability

The raw data supporting the conclusions of this article will be made available by the authors on request.

## Appendix A

**Table A1:** Classification criteria and basic characteristics of fire-severity treatments in the alpine forest study site.

Fireland Severity	Mean Scorch Height/Mean Height of Trees	Tree Mortality/%	Survival Status of Tree and Share Canopy
U	—	—	—
L	<1/3	<30	Most vegetation survived
M	1/3—2/3	30—70	Most of the shrub canopy was scorched, while a small proportion of the tree canopy survived
H	>2/3	>70	The shrub layer was completely burned, and most conifer needles in the tree canopy were completely charred

Notes: U, unburned; L, low fire severity; M, moderate fire severity; H, high fire severity. Same abbreviations apply throughout.

**Table A2:** Basic features of natural forest with different fire severities.

Fireland Severity	Longitude	Latitude	Canopy Density/%	Altitude/m	Dominant Herbaceous Species
U	101°13′5.8693″	30°12′38.4592″	65	2834	*Galinsoga parviflora*; *Artemisia sacrorum var. Messerschmidtiana*
L	101°13′6.3053″	30°12′0.5292″	60	2848	*Coptis chinensis*; *Artemisia sacrorum var.* Messerschmidtiana
M	101°13′6.9323″	30°12′02.4160″	30	2856	*Polygonum macrophyllum*; *Euphorbia fischeriana*
H	101°13′10.1823″	30°12′5.6327″	15	2864	*Eleusine indica*; *Achyranthes aspera*; *Nepeta cataria*

**Table A3:** Soil physicochemical properties under different fire-severity treatments.

Soil Physicochemical Properties	U	L	M	H
pH	6.559 ± 0.071 a	6.240 ± 0.268 a	5.888 ± 0.072 a	6.404 ± 0.150 a
EC (μs·cm^−1^)	49.667 ± 3.180 a	26.667 ± 1.333 b	30.667 ± 4.667 b	14.333 ± 1.202 c
SWC (%)	13.170 ± 0.580 d	18.223 ± 0.215 c	34.613 ± 0.249 a	31.837 ± 0.206 b
MWD (mm)	3.492 ± 0.253 a	2.936 ± 0.039 b	3.726 ± 0.036 a	2.915 ± 0.109 b
GMD (mm)	2.523 ± 0.084 b	2.143 ± 0.012 c	2.903 ± 0.116 a	2.233 ± 0.144 bc
AN (mg·kg^−1^)	1.195 ± 0.131 b	1.483 ± 0.083 a	0.883 ± 0.044 c	0.466 ± 0.050 d
AK (mg·kg^−1^)	489.205 ± 12.823 a	415.861 ± 0.482 b	490.090 ± 1.708 a	304.505 ± 3.468 c
NN (mg·kg^−1^)	5.323 ± 0.047 a	4.600 ± 0.174 a	4.863 ± 0.130 a	5.030 ± 0.322 a
AP (mg·kg^−1^)	8.887 ± 0.419 a	8.072 ± 0.056 ab	7.509 ± 0.092 b	3.894 ± 0.131 c
WSOC (g·kg^−1^)	0.420 ± 0.021 a	0.357 ± 0.038 ab	0.393 ± 0.024 a	0.243 ± 0.056 b
EOC (g·kg^−1^)	1.451 ± 0.045 a	1.504 ± 0.111 a	1.411 ± 0.061 a	1.395 ± 0.031 a
SOC (g·kg^−1^)	79.580 ± 2.641 c	48.470 ± 0.061 d	97.197 ± 1.075 a	91.400 ± 1.433 b
MBC (mg·kg^−1^)	752.529 ± 10.095 d	1056.465 ± 36.472 c	1166.265 ± 80.025 b	1256.520 ± 20.004 a
MBN (mg·kg^−1^)	58.510 ± 1.218 c	66.820 ± 0.415 b	84.150 ± 1.284 a	86.647 ± 2.545 a
MBP (mg·kg^−1^)	28.648 ± 1.221 b	28.917 ± 0.824 b	38.006 ± 1.312 a	39.996 ± 1.452 a

Notes: EC: soil electrical conductivity; SWC: soil water content; MWD: mean weight diameter; GMD: geometric mean diameter; AN: ammonium nitrogen; AK: available potassium; NN: nitrate nitrogen; AP: available phosphorus; WSOC: water-soluble organic carbon; EOC: easily oxidizable organic carbon; SOC: soil organic carbon; MBC: microbial biomass carbon; MBN: microbial biomass nitrogen; MBP: microbial biomass phosphorus. Different lowercase letters within the same group indicate a statistically significant difference of 0.05 between treatment groups.

**Table A4:** Redundancy analysis of dominant microbial genera and soil physicochemical properties.

Group	Soil Physicochemical Properties	Explains %	Contribution/%	F	*p*
Bacteria	AN	25.4	25.4	3.4	0.044
	SWC	22.2	22.2	3.8	0.048
	MBC	14	14	2.9	0.05
	MBP	10.7	10.7	2.7	0.05
	AP	7.2	7.2	2.4	0.124
Fungi	MBN	23.8	23.8	5.8	0.016
	AK	22.6	22.6	3.6	0.05
	MWD	20.7	20.7	2.6	0.066
	MBC	11	11	3.5	0.05
	MBP	4.7	4.7	1.6	0.21
